# A discrepancy between CT angiography and transesophageal echocardiographic measurements of the annular size affect long-term survival following trans-catheter aortic valve replacement

**DOI:** 10.34172/jcvtr.2021.39

**Published:** 2021-08-25

**Authors:** Siddarth Singh, Piotr S. Rutkowski, Alexey Dyachkov, Vijay S. Iyer, Leili Pourafkari, Nader D. Nader

**Affiliations:** ^1^Department of Anesthesiology, University of Southern California, Keck School of Medicine, Los Angeles, CA, USA; ^2^Department of Anesthesiology, University at Buffalo, School of Medicine and Biomedical Sciences, Buffalo, NY, USA; ^3^Department of Anesthesiology, Geisinger Medical Center, Danville, PA, USA; ^4^Gates Vascular Institute, Interventional Cardiology, University at Buffalo, School of Medicine and Biomedical Sciences, Buffalo, NY, USA; ^5^Catholic Health System, University at Buffalo Jacob’s School of Medicine and Biomedical Sciences, Buffalo, NY, USA

**Keywords:** Aortic Stenosis, Computed Tomographic, Angiography, Echocardiography, Trans-catheter Aortic Valve, Replacement

## Abstract

***Introduction:*** Accurate measurement of the aortic valve annulus is critical for proper valve sizing for the transcatheter aortic valve replacement (TAVR) procedure. While computed tomography angiography (CTA) is the widely-accepted standard, two-dimensional (2D) and three-dimensional(3D) transesophageal echocardiography (TEE) is commonly performed to measure the size of the aortic valve and to verify appropriate seating of prostheses.

***Methods:*** Patients undergoing TAVR between 2013-2015 were examined. 2D- and 3D-TEEmeasurements were compared to CTA taken as standard. Patients were followed for at least one year. The presence and effect of discrepancy (defined as a difference of more than 10%) between CTA and TEE measurements on survival were examined.

***Results:*** One hundred eighty-five patients (70 men) were included. 2D- and 3D-TEE measurements underestimated the annulus size by -1.49 and -1.32 mm, respectively. Discrepancies > 10% between TEE and CTA methods in estimating the aortic annulus size were associated with a decrease in post implant survival. The peak pressure gradient across the aortic prosthesis measured one year after the implant was higher in patients with an initial discrepancy between 3D-TEE and CTA measurements. In a multivariate cox-regression model, the discrepancy between CTA and 2D-TEE readings and the smaller size of the aortic annular area were the predictors of long-term survival.

***Conclusion:*** Both 2D and 3D-TEE underestimate the aortic annulus measurements compared to CTA, with 2D-TEE being relatively more precise than 3D-TEE technology. The presence of a discrepancy between echocardiographic and CTA measurements of the aortic annulus is associated with a lower survival rate.

## Introduction

The rapid evolution of transcatheter aortic valve replacement (TAVR) and technology has pushed for likewise evolution in cardiac imaging techniques and interpretation, mainly focusing on the size of the aortic valve annulus. This single measurement has become critical in the proper sizing of prosthetic transcatheter valves. It has fueled several recent studies in determining the most appropriate imaging modality and measuring techniques concerning the accuracy and prognostic predictability in terms of prosthesis mismatch and regurgitation.^1-3^ According to the American Society of Echocardiography guidelines published in 2011, there is no gold standard in measuring aortic valve annulus.^4^

Tsang et al found that cardiac magnetic resonance imaging (CMRI) measurements had the highest accuracy and lowest variability, followed by multi-sliced computed tomographic angiography (CTA).^5^ Compared to CMRI technology, the CTA method overestimated and three-dimensional (3D) transesophageal echocardiography (TEE) underestimated the annulus area’s actual size. Although CMRI appears to be the actual gold standard,^6^ it is not routinely used. Instead, CTA has become the widely-accepted standard, largely replacing two-dimensional echocardiography (2D-TEE) due to the complex nature of the aortic annulus’ geometry.

Both 2D-TEE and 3D-TEE are acceptable methods; however, more significant limitations have been placed on the ability to correctly size the annulus based on 2D-TEE because of the 3D coronet geometry of the aortic annulus, which is more oval or elliptically shaped, especially in the elderly population.^7,8^ The irregular geometry is further complicated and distorted by disease and often severe calcifications. Since 3D-TEE provides views of the aortic valve diameter in both coronal (maximal) and sagittal (minimal) sections,^9^ it allows more detailed visualization of anatomy at the base of the aortic valve hinge points. Therefore, 3-D TEE imaging is theoretically approaching the accuracy of CTA imaging in current practice.

Our institution has experienced a rapidly increasing volume of referrals for transcatheter aortic valve replacement (TAVR) procedures with increasing reliance on routine imaging studies. Currently, all valves are sized based on CTA with 2D-TTE first taken into consideration as well. All patients who receive general anesthesia also have a full TEE exam performed, which includes 3D measurement of the aortic annular circumference during systole, but these measurements are not used for selecting the valve prosthesis size. This study aimed to examine the discrepancy between 2D-TEE and 3D-TEE with the widely-accepted standard of CTA. We evaluated whether the discrepancy between 3D-TEE and CTA measurements could affect patients’ one-year outcome and overall survival. Our null hypothesis is that there is no significant difference between aortic valve annulus dimensions when comparing 2D-TEE and 3D-TEE techniques to the CTA method.

## Materials and Methods

This work was a single-center cohort study on data collected from the perioperative imaging studies of the patients undergoing TAVR followed by a longitudinal follow-up for the occurrence of valve-related complications and overall survival. Study design and protocol were reviewed and approved by the Institutional Review Board of the State University of New York at Buffalo. The study was waived from obtaining informed consent due to its non-invasive nature.

From 2010 to 2012, all patients undergoing TAVR at our institution were sized for aortic valve prosthesis using CTA. All patients also had preoperative transthoracic echocardiography (TTE) exams. CTA was primarily used for valve sizing. 2D measurements were obtained from TTE were not included in this study due to the inability to verify the measurement technique. Patients were excluded if they had a prior aortic valve replacement. The CTA was read by a radiologist specialized in cardiovascular imaging and was reported as average diameter/circumference in systole. Furthermore, we excluded six patients from outcome analysis due to missing one or more modalities of measurements for the aortic annulus size.

Intra-operatively, those patients who underwent general anesthesia for the procedure had a complete TEE exam performed by one of three cardiologists proficient at 3D image acquisition using iE33 echocardiography machines (Phillips®, Andover, Massachusetts). 2D aortic annulus size was reported in diameter and was measured at the aortic valve hinge points in the standard aortic valve long-axis view. 3D images were obtained after acquiring a full volume 3D image and using 3DQ software. This software allows the cardiologist to align three multiplane reconstruction reference planes (MPRs), sagittal, coronal, and transverse, to obtain an optimal view of the aortic valve annulus. Subsequently, the annulus area was traced manually using a curser to achieve a measured area and the circumference. If multiple measurements were obtained, these were averaged.

Data from these patients were gathered using the electronic medical record and reading all radiology and cardiology imaging reports, and viewing all echocardiographic images to verify the method and consistency of technique. Mortality data were also gathered by obtaining obituary records of patients. The aortic annular area was traced using either CTA or 3D-TEE imaging modalities, and the diameter was then mathematically calculated. All measurements were made during systole. TEE dimensions were not used to influence the valves’ sizing, which had been already preselected based on the CTA study. The discrepancy was defined as a difference of more than 10% between the TEE and CTA annulus size measurements.

### Statistical analysis

All data obtained in this study are continuous and represented as mean and standard deviation. The level of agreement between two continuous variables was assessed using Bland-Altman plots.^10^ A one-sample t-test determined proportional bias in the measurement and a correlation coefficient using linear regression scatter plots. We considered CTA as the gold standard and compared each echocardiographic modality to CTA independently. The effect of a discrepancy between CTA and TEE measurements on survival was examined. Differences were deemed statistically significant at p values less than 0.05. Kaplan-Meier plot analysis was done to determine the survival function. Following multiple univariate analyses, all factors with near significant effect (p values ≤ 0.1) on the primary time-to-event endpoint (post-TAVR mortality) were entered into a Cox regression model for multivariate analysis. Hazard risk ratios for these factors were calculated, and the significance with *P* values < 0.05 was considered an independent predictor of mortality.

## Results

From 185 TAVR patients (70 males and 115 females), only 179 had complete periprocedural imaging studies. The average age of 83.9  ±  4.9 years old was reviewed. All patients had received general anesthesia and underwent TAVRs using trans-femoral, trans-aortic, or trans-apical approaches. The traced aortic annulus area was 4.45  ±  0.87 cm^2, and the average diameter was calculated at 23.7  ±  2.4 mm by CTA imaging. The mean aortic diameter measured at the aortic valve hinge points in the standard aortic valve long-axis view of 2D-TEE was 22.2  ±  2.3 mm, and 3D-TEE was 22.4  ±  2.5 mm. Seventy-five out of 185 patients died during the 4-year follow-up period. A great majority (92%) of the deceased patients died to exacerbate their underlying cardiac condition or resulting systolic heart failure. Clinical, anatomic, and demographic characteristics of survivors and deceased patients are tabulated in Table 1.

**Table 1:** Patient characteristics and imaging information in groups of patients with respect to the occurrence of death within 42 months of the post-procedural follow-up period.

		**Survivors (N=110)**	**Non-survivors (N=75)**	**OR (95% CI)**	***P*** ** Value**
Gender	Female	65 (59.1%)	50 (66.7%)	0.722 (0.39-1.33)	0.203
	Male	45 (40.9%)	25 (33.3%)		
Age (year)		83.2 ± 4.8	85.4 ± 5.0	1.100 (1.03-1.17)	0.003
Functional NYHA Class	I/II	101 (91.8%)	69 (92.0%)	0.976 (0.33-2.87)	> 0.999
	III/IV	9 (8.2%)	6 (8.0%)		
Aortic Insufficiency (> mild) *		15 (16.9%)	13 (24.5%)	1.233 (0.78-1.93)	0.385
Heart Failure © reduced EF		24 (27.0%)	15 (28.3%)	1.069 (0.50-2.28)	0.404
Heart Failure © preserved EF		68 (76.4%)	26 (52.0%)	0.335 (0.16-0.71)	0.004
LV Ejection Fraction (%)		52.4 ± 15.9	58.4 ± 10.6	1.034 (1.00-1.06)	0.007
Valve type	Core Valve	30 (27.3%)	22 (29.3%)		0.120
	Sapien XT	74 (67.3%)	53 (70.7%)		
	Lotus Edge	6 (5.5%)	0 (0.0%)		
Effective Orifice Area (cm^2^)		1.96 ± 0.31	1.83 ± 0.47	0.302 (0.11-0.84)	0.037
Annulus size on CTA (mm)		23.7 ± 2.4	23.7 ± 2.3	1.001 (0.88-1.14)	0.990
	Annulus Size with 2D Echo (mm)	22.5 ± 2.5	21.7 ± 1.7	0.818 (0.70-0.96)	0.012
Annulus Size with 3D Echo (mm)		22.8 ± 2.4	21.7 ± 2.6	0.822 (0.72-0.94)	0.003
Peak gradient (mm Hg) Post-TAVI		14.2 ± 6.4	17.8 ± 11.2	1.049 (1.01-1.09)	0.027
Mean gradient (mm Hg) Post-TAVI		7.5 ± 3.7	9.6 ± 6.5	1.067 (0.99-1.15)	0.052

Abbreviation: NYHA, New York Heart Association; EF, Ejection Fraction; TAVI,: Trans-catheter Aortic Valve Implant; CI, confidence interval; CTA,computed tomography angiography

**Figure 1:**
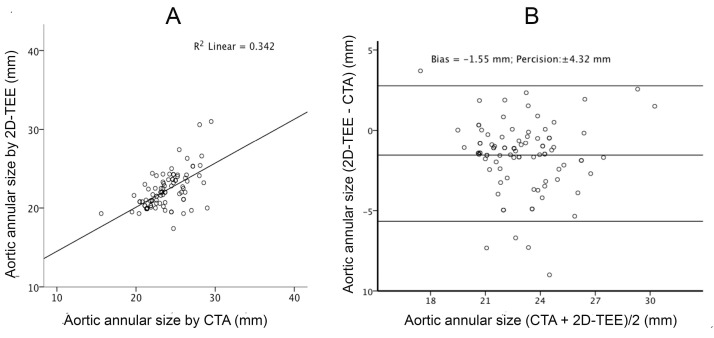
Scatterplot (A) depicting a linear correlation between the aortic annular size measurements obtained 2-dimensional transesophageal echocardiography (2D-TEE) and computerized tomographic angiography (CTA). Bland-Altman plot (B) on the right examines the bias and precision of aortic annular diameter measurement by 2D-TEE compared to CTA as the gold standard.

**Figure 2:**
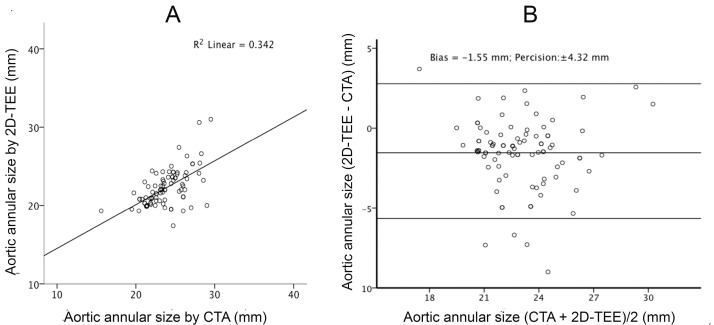
Scatterplot (A) depicting a linear correlation between the aortic annular size measurements obtained 3-dimensional transesophageal echocardiography (3D-TEE) and computerized tomographic angiography (CTA). Bland-Altman plot (B) on the right examines the bias and precision of aortic annular diameter measurement by 3D-TEE compared to CTA as the gold standard.

There was a linear correlation between CTA and 2D-TEE with R^2 of 0.314 (*P* <  0.001; Figure 1A). In Bland-Altman analysis, the mean values between the CTA and 2D-TEE measurements were plotted against the difference between 2D-TEE and CTA. The calculations showed that 2D-TEE measurements underestimated the annulus size by a bias of -1.49 mm with a level of agreement of  ±  4.23 mm (  ±  1.96 * standard deviation) (Figure 1B). Similarly, there was a linear correlation between CTA and 3D-TEE with an R^2 value of 0.325 and *P* value < 0.001 (Figure 2A). The measured bias between CTA and 3D-TEE in measuring the size of aortic annulus diameter was -1.32 mm with a level of agreement (precision) of  ±  4.45 mm (Figure 2B). Although the underestimation of the annulus size was less with 3D-TEE, the measurement’s precision was greater than 2D-TEE, and the linear correlation to the CTA method was accordingly higher.

From 179 patients, there was a discrepancy of > 10% between 2D-TEE and CTA measurements in 40 patients. Similarly, the 3D-TEE measurements differed > 10% against the CTA readings in estimating the diameter of the aortic annulus of 19 patients. The patients’ demographics and characteristics are tabulated in Table 2 according to the> 10% discrepancy of two echocardiographic modalities concerning CTA. Male gender was more common among the patients, with a more significant discrepancy between TEE and CTA methods. There was no difference in the patients’ age with or without discrepancy in the two sizing methods. The choice of valve implants was not affected by the presence of discrepant readings. Additionally, the baseline functional class and the left ventricular ejection fraction were similar between the two groups.

**Table 2:** Patient characteristics and imaging information in groups of patients with respect to the presence of  ±  10% discrepancy in CT Angiographic measurements of the aortic annulus and those measured by 2-dimensional and 3-dimensional transesophageal echocardiography

		**(-) Discrepancy CTA vs. 2D-TEE** **(N=139)**	**(+) Discrepancy CTA vs. 2D-TEE** **(N=40)**	***P*** ** value**	**(-) Discrepancy CTA vs. 3D-TEE** **(N=147)**	**(+) Discrepancy CTA vs. 3D-TEE** **(N=32)**	***P*** ** value**
Female Gender		91 (65.5%)	22 (55.0%)	0.266	100 (68.0%)	13 (40.6%)	0.005
Male Gender		48 (34.5%)	18 (45.0%)		47 (32.0%)	19 (59.6%)	
Age (years)		84.1 ± 5.2	83.7 ± 4.5	0.647	84.0 ± 5.2	83.8 ± 4.3	0.823
Type (CV/ES/LT)		39 / 94 / 6	13 / 27 / 0	0.380	42 / 99 / 6	10 / 22 / 0	0.502
Effective Orifice Area (cm^2^)		1.90 ± 0.39	2.00 ± 0.37	0.364	1.92 ± 0.40	1.86 ± 0.31	0.412
Aortic Diameter by CTA (mm)		23.3 ± 2.0	25.0 ± 3.1	0.001	23.3 ± 2.4	25.2 ± 1.9	< 0.001
Aortic Diameter by 2D-TEE (mm)		22.6 ± 2.2	20.9 ± 1.7	< 0.001	22.4 ± 2.2	21.2 ± 2.1	0.004
Aortic Diameter by 3D-TEE (mm)		22.7 ± 2.1	21.2 ± 3.4	0.008	22.8 ± 2.3	20.5 ± 2.7	< 0.001
Aortic Insufficiency (Mild)		22 (15.8%)	0 (0.0%)	0.005	22 (18.8%)	0 (0.0%)	0.015
NYHA Class	I / II	124 (89.2%)	40 (100%)	0.025	134 (91.2%)	30 (93.7%)	> 0.999
	III / IV	15 (10.8%)	0 (0.0%)		13 (8.8%)	2 (6.3%)	
Systolic Heart Failure		31 (27.0%)	2 (9.5%)	0.102	31 (26.5%)	2 (10.5%)	0.160
Diastolic Heart Failure		79 (70.5%)	15 (71.4%)	1.000	81 (71.1%)	13 (68.4%)	0.791
LV Ejection Fraction (%)		54.4 ± 15.5	58.5 ± 7.2	0.058	54.9 ± 15.5	56.1 ± 6.6	0.573
Peak Gradient in FU (mm Hg)		14.4 ± 7.2	20.9 ± 12.2	0.028	14.7 ± 7.8	20.0 ± 10.8	0.050
Mean Gradient in FU (mm Hg)		8.0 ± 4.6	9.8 ± 6.1	0.113	8.0 ± 4.7	9.8 ± 5.8	0.141
Mortality in one year		11 (7.9%)	6 (15.0%)	0.219	9 (6.1%)	8 (25.0%)	0.003
Overall Death Rate		43 (30.9%)	26 (65.0%)	< 0.001	46 (31.3%)	23 (71.9%)	< 0.001

Abbreviation: NYHA, New York Heart Association; CTA, computed tomography angiography;TEE,tranesophageal echocardiography, CV/ES/LT, Corevalve/ Edwards Sapien/Lotus;LV, left ventricle;FU, follow up

Clinical and echocardiographic characteristics of the implanted valves were examined within one year of the procedure. Peak transvalvular gradient was significantly higher in patients with > 10% discrepancy between 3D-TEE and CTA measurements of the aortic annular size (21.3  ±  13.1 vs. 15.0  ±  7.9 mmHg; *P* = 0.048). A similar observation was not seen in patients with discrepant readings between 2D-TEE and CTA. (Table 2) The one-year mortality rate was significantly higher in the 3D-TEE discrepant group than the non-discrepant group (*P* = 0.017), while the difference was not significant for 2D-TEE.

**Figure 3:**
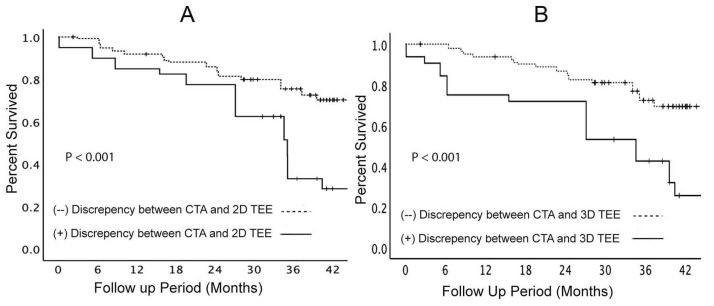
Panel A depicts Kaplan-Meier's analysis of the patients grouped based on the presence of discrepancy in measuring aortic annular diameter by 2-dimensional transesophageal echocardiography (2D-TEE) CTA. Panel B depicts Kaplan-Meier analysis of the patients grouped based on the presence of discrepancy in measuring aortic annular diameter by 3-dimensional transesophageal echocardiography (3D-TEE) and CTA.

**Figure 4:**
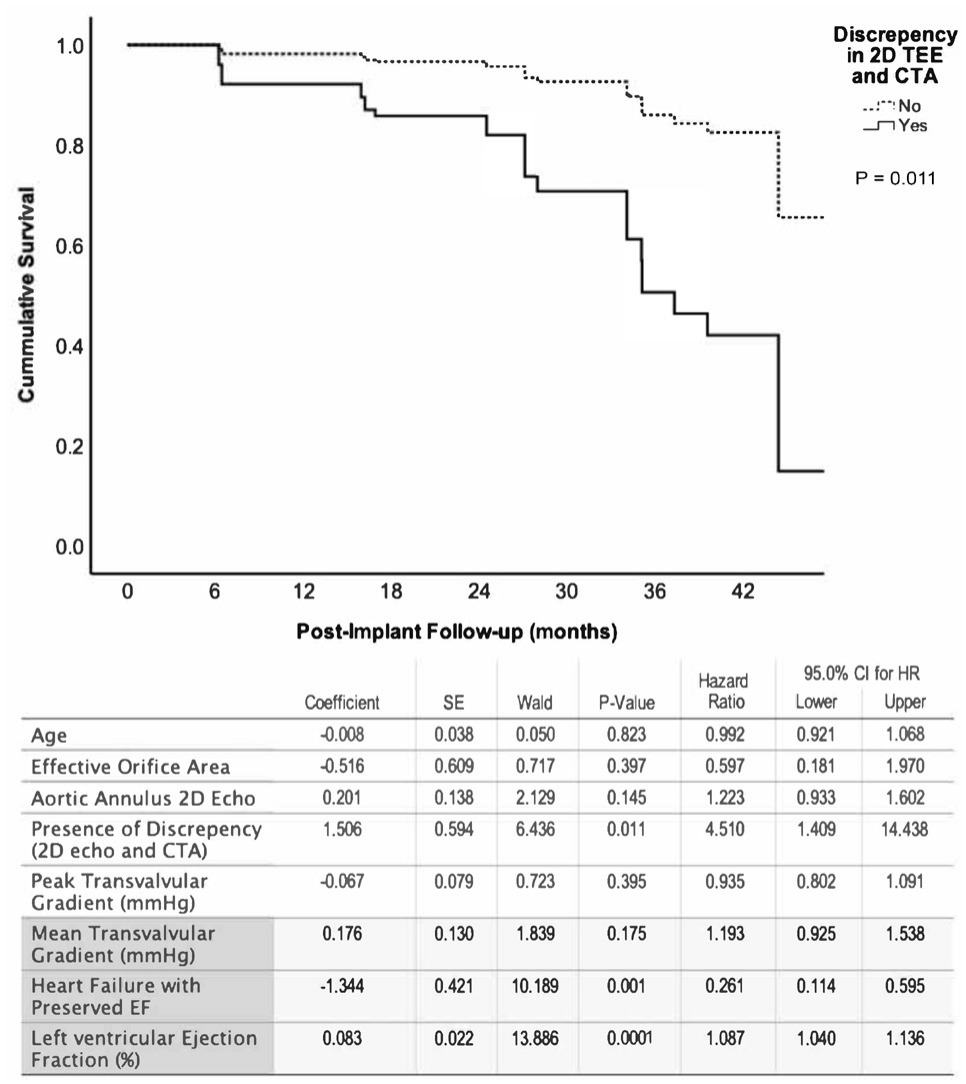
Multivariate Cox regression model that examines the role of a discrepancy between CTA and 3D-TEE measurements of the aortic annulus over 42 months of follow-up period after TAVR. All variables that had historical significance or were found to have a significant effect on mortality by a univariate analysis have been included in this model.

**Figure 5:**
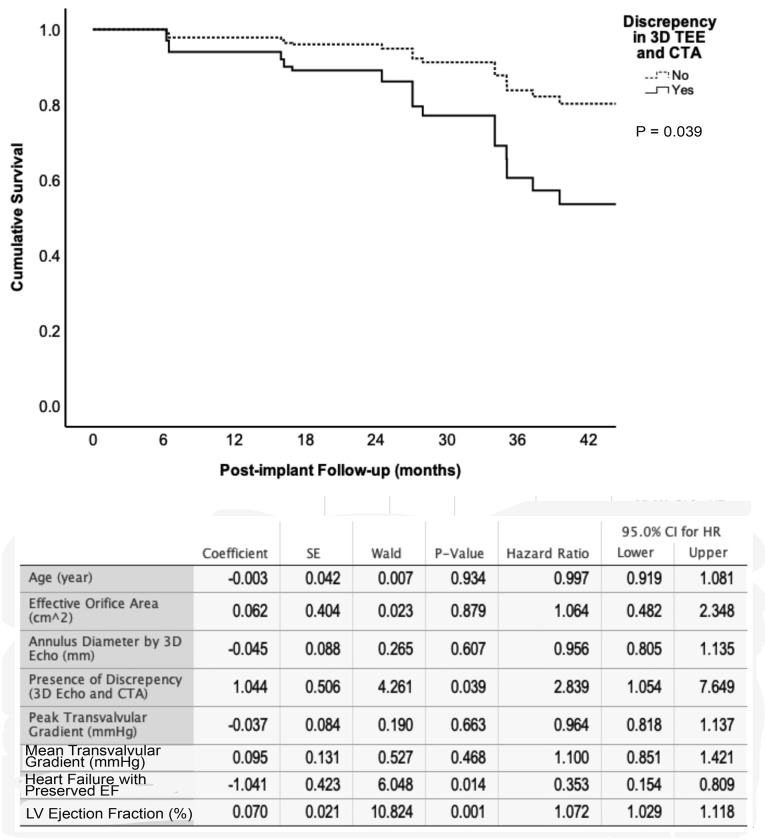
Multivariate Cox regression model that examines the role of a discrepancy between CTA and 3D-TEE measurements of the aortic annulus over 42 months of follow-up period after TAVR. All variables that had historical significance or were found to have a significant effect on mortality by a univariate analysis have been included in this model.

The clinical impact of the presence of discrepancy was measured by examining the mortality difference over a 42-month period for patients with and without discrepancies with the CTA measurement. The overall univariate analyses of survival rates for patients in either (2D-TEE vs. CTA analyses; Figure 3A) or (3D-TEE vs. CTA analyses; Figure 3B) were negatively affected if there was > 10% reading discrepancy in the size of the aortic annulus. Consequently, a multivariate cox regression model constructed to examine the role of the confounding factors on patient survival showed that the difference between CTA and 2D-TEE to be the only independent factor predicting overall survival with a p-value of 0.011 (Figure 4) in addition to the baseline annular area measured by CTA. Additionally, the multivariate regression model was constructed by forcing the discrepancy > 10% between 3D-TEE and CTA of the aortic annulus size, which demonstrated a better survival of those without such a discrepancy after TAVR (Figure 5).

## Discussion

Our findings support the previously reported relationships between measurements obtained by 2D-TEE, 3D-TEE, and CTA^11,12^ Consistent with these studies, both echocardiographic modalities were significantly underestimated compared to those measured by multi-sliced CTA imaging. Overall, diameters derived from 3D-TEE area tracing were more extensive than the 2D measurement of the aortic annular diameters. The annulus’s elliptical geometry with the minimum diameter in the sagittal plane accounts for the underestimation using the 2D method, which assumes that the annulus has a perfect circular shape.^13^ Intraoperative role of TEE is increasing during TAVR procedures for verification of appropriate size and proper seating of the prosthetic valves.^14^ This study serves to provide a snapshot of the clinical experience to date in a field that is still rapidly evolving, and it cannot be interpreted to support one imaging modality over another.

In a prospective multi-center study, incorporating annulus area sizing through multi-slicing CTA was associated with less paravalvular regurgitation.^15^ The routine use of CTA has its risks and limitations, especially in the TAVR population may not tolerate heart rate control or intravenous contrast use due to poor renal function. Several recent studies have made comparisons between 2D and 3D imaging. In a recent report by Guez et al 3D-TEE and CTA aortic annular measurements of 74 patients were retrospectively compared.^16^ These authors reported a too high correlation (R-value of 0.91) between the two techniques. Although still significant, the correlation between 3D-TEE and CTA measurements of the aortic annulus was more modest in our report.

In another study that had compared multi-sliced CTA with 2D-TTE and 3D-TEE in 45 patients, mean differences of 1.22 mm and 1.52 mm were reported, respectively, with a higher correlation between 2D-TTE and 3D-TEE.^17^ The correlation between the two echocardiographic techniques was higher than the correlation of either modality with CTA. These investigators recommended using perioperative TEE to estimate prosthetic valve size as it was associated with good clinical results. In a study of 49 patients, with a mean difference of 1.22 mm, 3D-TEE overestimated the aortic annulus’ size compared to 2D-TTE. Such a difference in annular sizing was significant and impacted the selection of prosthesis size in clinical practice.^11^ Comparably, in our patient pool, the correlation between the two echocardiographic modalities was robust (R-value of 0.747), which was significantly higher than their correlation with CTA measurements.

Although 2D-TTE is still an accepted modality, studies have revealed that the elliptical annulus’s minimal diameter is in the sagittal plane, measured in the long-axis view of the aortic valve, therefore significantly underestimating the annular size. The value of 3D over 2D echocardiography has also been shown with studies comparing the two concerning clinical outcomes when these methods are used for valve size selection. The use of 3D imaging during TTE is not recommended due to the lower quality of the images. As TEE is the best imaging modality for 3D imaging, it is gradually replacing 2D imaging due to its inherent geometric limitations. As technicians and cardiologists become more familiar with the 3D technology and ability to optimize the annulus view and trace it appropriately, one can anticipate that future data will become more reproducible and representative of the actual difference between the 2D and 3D techniques. Meanwhile, novel 3D techniques are emerging which may offer higher accuracy and utility in clinical practice.^2^

Jilaihawi et al found that 3D measurements were significantly superior to 2D-TEE concerning post-TAVR paravalvular aortic regurgitation.^3^ Another study found a significantly higher incidence of severe patient-prosthesis mismatch after TAVR where 2D-TTE and 3D-TEE sized the implanted valves.^1^ The evidence so far is based on a minimal number of studies and low sample sizes comparing these two modalities. In general, the results favor 3D over 2D in terms of accuracy and prognostic predictability. Our findings suggest that 2D-TEE offered a better precision than the 3D-TEE while it had a higher bias than CTA measurements. However, the discrepancy of > 10% between 3D-TEE and CTA measured diameters was associated with a higher trans-valvular peak gradient, higher one-year death rate, and worse overall survival. Even after controlling for all other factors such as age, effective orifice size of the implanted valve, aortic insufficiency, transvalvular gradients, the presence of systolic heart failure, and ejection fraction, the discrepancy between CTA and 3D-TEE was associated with a worse outcome (factors included in multivariate analyses in Figure 5).

A better understanding of the causes that may lead to a discrepancy in sizing is also a matter of interest. A greater degree of calcification over the aortic valve deteriorates the quality of the echocardiographic images and makes it difficult to obtain accurate measurements. Echocardiographic determination of the annular diameter of the aortic valve involves measuring intertrigonal distance that generally measured the internal diameter of the anatomic structure. In contrast, CTA measurements are based on the determination of the outer circumference/diameters. Therefore, it is to assume that the aortic wall thickness contributes to the most discrepancy in measurement. The thicker aortic wall correlates to the more considerable difference between the diameter measured by echocardiography and CTA’s diameter. We speculate that the thickness of the aortic wall may also contribute to the shorter overall survival of the patients than those in whom the aortic wall is not excessively thickened.

The presence of a greater amount of calcium could have also contributed to the worsened outcome in patients with discrepant aortic diameter measurements. The degree of valvular deposits of calcium could also contribute to paravalvular leak due to the greater degree of irregularity leading to paravalvular leak and secondary heart failure. Higher grades of calcification may be attributed to hypertension due to the existing shearing forces, exuberant proinflammatory responses, and excessive oxidative injury of the valve (rheumatic diseases).^18^ Estrogens possess inhibitory effects on the aortic annulus’ calcium formation through suppressing receptor activator of nuclear factor kappa-B ligand (RANKL) signaling^19^, thereby providing an explanation to a relatively higher frequency of valvular calcification in men than in women with aortic stenosis.^20^ In our study, we have found a higher level of discrepancy in measuring aortic annular size in men than in women. The gender differences in the degree of calcification may justify this finding.

The greatest limitation of this study is that it is a cohort study that spans a period. Therefore, it certainly involves a learning curve for all involved parties, including interventional cardiologists, radiologists, cardiac anesthesiologists, and non-interventional cardiologists. Although ultrasonographers and cardiologists at this institution are well versed in 3D image acquisition and interpretation, this study does not account for the nuances that may exist in technique and deviation from guidelines. Due to the varying levels of experience among physicians with 3D imaging, we certainly expect more significant intra- and inter-observer variabilities, which were not analyzed in this study. Likewise, we expect that there may be significant inter-observer variability in CTA measurements of the aortic annulus. Also notable is the varying level of calcium burden in these patients, which may affect measurement accuracy by 3D, and inter-observer interpretation of the blood-tissue interface. Because calcium produces shadowing artifacts and interferes with the ultrasound image, CTA images provide a more apparent distinction between blood and tissue, which may account for the underestimation by 3D. Lastly, a significant limitation to this study is the temporal difference in aortic annulus measurements and the level of sedation of the patient. Hemodynamic and loading conditions are expected to distort the annulus size, especially under general anesthesia. Further, the precise time frame of systole (early, mid, or late), which the measurement was taken, may have confounded the data.

## Conclusion

We concluded that the discrepancy between measuring outer (adventitial) diameter/area and inner (intimal) diameter/area of the aorta is associated with lower survival after TAVR procedures; this may be due to the increased wall thickness or the presence of excessive calcification over the annulus of the aorta that both may contribute to an unfavorable outcome in TAVR patients. The predictive role of these risk factors needs to be validated through larger prospective studies.

## Acknowledgments

None.

## Competing interest

The authors report no financial relationships or conflicts of interest regarding the content herein.

## Ethical Approval

The study was reviewed and approved by the Institutional Review Board at the University at Buffalo, School of Medicine and Biomedical Sciences for its ethical and scientific merit.

## Funding

No funding was requested.
